# Breastfeeding Support in the Early Postpartum: Content of Home Visits in the SILC Trial

**DOI:** 10.1111/birt.12241

**Published:** 2016-07-15

**Authors:** Lael Ridgway, Rhian Cramer, Helen L. McLachlan, Della A. Forster, Méabh Cullinane, Touran Shafiei, Lisa H. Amir

**Affiliations:** ^1^ School of Nursing & MidwiferyLa Trobe UniversityBundooraVic.Australia; ^2^ Judith Lumley CentreLa Trobe UniversityMelbourneVic.Australia; ^3^ School of NursingMidwifery and HealthcareFederation UniversityMt HelenVic.Australia; ^4^ Judith Lumley Centre & School of Nursing & MidwiferyLa Trobe UniversityMelbourneVic.Australia; ^5^ The Royal Women's HospitalParkvilleVic.Australia

**Keywords:** breastfeeding, child and family health, community, nursing, professional lactation support

## Abstract

**Background:**

The Supporting breastfeeding In Local Communities (SILC) cluster randomized controlled trial evaluated whether a home visit focussed on infant feeding early in the postpartum period increased the percentage of infants breastfeeding at 4 months in low socioeconomic areas of Victoria, Australia. The visits were offered to women identified as at risk of early breastfeeding cessation after discharge home with a new baby. This paper describes the content of the home visits.

**Methods:**

SILC visited 1,043 women from September 2012 to March 2013, and completed a data sheet for each visit documenting topics discussed, as well as what support and resources were offered. Frequencies and percentages are presented.

**Results:**

Home visits most commonly included the provision of reassurance to women (91%). Topics discussed included general breastfeeding information (83%), supply and demand (83%), positioning (79%), and feeding frequency (78%). Newborn feeding/behavior (57%), expression of breastmilk (54%), nipple pain (41%), low breastmilk supply (41%), and the use of nipple shields (18%) were also prominent topics. The issues and support needs of women were similar across locations (rural, regional or metropolitan) and regardless of maternal parity or age. There was some variation in the resources suggested in different localities.

**Conclusions:**

New mothers require help and reassurance independent of whether this is their first or subsequent child, reinforcing the need for support, breastfeeding information, and education about normal neonatal behavior. Key aspects of support are reassurance, normalization of infant behavior, and education.

Overall, Australia has very high breastfeeding initiation, but there is a sharp decline in the first weeks after birth and this continues throughout the first year after birth 1, 2. While 96 percent of Australian women initiate breastfeeding 2, in the state of Victoria, only 50 percent of infants on average are receiving *any* breastmilk at 6 months of age 3.

It is recognized that new mothers need to feel confident and supported in the early stages of their breastfeeding experience 4, 5, 6, 7, 8, and first‐time mothers have been found to progress from being “on shaky ground” through to gaining more information and understanding to eventually being “at ease” with breastfeeding 4. A 2012 Cochrane review of breastfeeding support identified early support for breastfeeding, including both professional and lay support, as having a positive influence on breastfeeding duration, with face‐to‐face support being the type most likely to succeed 5.

Supporting women to breastfeed through the introduction of peer and professional support in community programs is encouraged locally and internationally 6, 9, 10, 11, 12, but there is little information available about the concerns breastfeeding women have in the early days after discharge from maternity services. This knowledge is important if interventions to support and increase breastfeeding are going to address women's own concerns.

The Maternal and Child Health (MCH) service in Victoria, Australia provides universal, community‐based care to families with children under school age 10. It is provided by 79 municipalities with the additional support, funding, and oversight of the state government. Legislation requires that municipalities are notified of all births in their area. Contact from the MCH service is made with families as soon as possible after the municipality has been notified of a birth, usually within 1 week of discharge from hospital, to arrange a visit to the family's home. This visit usually takes place when the infant is between 8 and 12 days old. The initial in‐home consultation is followed by a series of scheduled center visits. These routine consultations are carried out by Maternal and Child Health Nurses (MCHNs): registered nurses who also hold midwifery and postgraduate child and family health qualifications. Additional services are provided to those families identified as having particular needs, through the Enhanced MCH service. Twenty‐four hour professional telephone support is also available 10.

The SILC (Supporting breastfeeding In Local Communities) trial was a cluster randomized controlled trial which aimed to increase breastfeeding rates by implementing two new community‐based interventions designed to provide breastfeeding support in the home during the early postpartum period to women at increased risk of early breastfeeding cessation 13. The trial was conducted in ten municipalities where breastfeeding rates were lower than the state average. Municipalities were randomized into one of three trial arms: a comparison arm (standard MCH care) and two intervention arms; early home‐based professional breastfeeding support; or access to a community‐based breastfeeding drop‐in center in addition to early home‐based professional breastfeeding support 13. These interventions occurred in addition to the routine MCH home visits described above.

Randomization was achieved using sealed envelopes, each containing a municipality name. A self‐selected independent audience member (not linked to any of the SILC‐participating municipalities) shuffled the envelopes and then randomly selected and opened individual envelopes to reveal the municipality. Municipalities were in turn assigned to the comparison arm, the SILC‐MCHN home‐visiting arm, and the SILC‐MCHN home‐visiting plus drop‐in center arm. This process was repeated until all municipalities were assigned to a trial arm 13.

Overall the SILC trial found no difference in the percentage of women providing any breastmilk to their infant at 4 months of age 13. While the women who received support valued it highly, far fewer women than anticipated actually received early home‐based breastfeeding support 13, something which might have contributed to the finding of no differences between trial arms.

This paper describes the SILC home visits and the breastfeeding issues and concerns addressed during these visits. Previous studies have largely focussed on women who themselves seek breastfeeding support services 14, 15, 16. These women are more likely to have a strong desire to breastfeed and have the confidence and motivation to overcome any issues encountered 17, 18. Because these SILC home visits were proactive and preventative, conducted in women's own homes and offered to all women identified as at risk of early cessation of breastfeeding, this paper addresses a gap in the literature by including a wider population of breastfeeding mothers, instead of only those who are motivated to seek out help independently. In addition, the paper also reports on the supports discussed with the women, including information provided and recommended resources and referrals, details of which are not available in the published literature.

## Methods

### SILC Intervention

The participant municipalities included rural, regional, and metropolitan communities. The number of infants receiving care in individual municipalities ranged from 469 to just over 4,000 infants annually in 2012–13 19, 20, 21, 22. Of the 10 SILC municipalities, and in conjunction with standard MCH care, home‐based breastfeeding support was provided in six. This included three with home‐visits (one each of rural, regional, and metropolitan) and three with visits and drop‐in center access (one each of rural, regional, and metropolitan). The six municipalities delivering the home‐visiting intervention are referred to as “Municipality 1” to “Municipality 6” in this paper.

Women were identified by telephone for eligibility for a SILC home visit as soon as possible after discharge from hospital. It was standard practice for municipalities to contact women by phone when they received notification of the birth. As far as possible, this eligibility assessment was incorporated into standard practice. Eligibility criteria were based on two of the early postnatal factors known to be associated with early breastfeeding cessation: infants who were receiving any infant formula or expressed breastmilk in the previous 24 hours 13. Visits were also offered if the woman was distressed at the time of the intake phone call, or if the center MCHN identified a need at a later routine visit and made a referral. It was planned (and expected) that 30 percent of women in the home‐visiting trial arms would have an average of two visits each 13.

During the trial, dedicated MCH nurses (SILC‐MCHNs) were employed, usually from within the municipality's existing MCH team, and funded by the Victorian State Government as part of the trial. These SILC‐MCHNs were experienced in providing breastfeeding support. Most were currently or previously certified International Board Certified Lactation Consultants. In all, 13 SILC‐MCHNs were recruited and trained to participate.

To ensure adherence to the trial protocols, SILC‐MCHNs attended workshops during the trial period. The first workshop, before commencement of the intervention, provided an introduction to the trial and research processes, as well as a breastfeeding management update. SILC‐MCHNs were encouraged to follow a proactive, family‐centered approach to providing breastfeeding support, based on recommendations by Hoddinott et al, who investigated the provision and success of breastfeeding support in predominantly deprived areas 23. Later workshops provided an opportunity for the SILC‐MCHNs and the research team to review processes and discuss any concerns.

The SILC‐MCHNs were asked to focus on breastfeeding during the home visit, and to address any concerns identified by the woman. Where appropriate, the SILC‐MCHNs also developed an infant feeding plan, provided information, and suggested resources such as websites and peer support groups. Referrals were made to other health care providers and additional breastfeeding services if necessary.

### Data Collection and Analysis

A precoded data collection form was developed for the SILC trial and provided to the SILC‐MCHNs for completion at the home‐visit (see Appendix 1, available as supporting information in the online version of this article). This form recorded: topics discussed during the home visit; referrals to other services; and information given to the women, including recommended fact sheets and websites 13. Infant feeding data were collected by asking about infant feeding “in the past 24 hours.” The data collection form was refined during the trial 2‐month run‐in period from July to August 2012. Data were entered into an Access database, cleaned using range and logic checks, and analyzed, using Stata 11 24.

We have summarized the data using descriptive statistics, and present outcomes in tables that enable comparison across municipalities, as well as some outcomes by maternal parity and age. Open‐ended comments were recoded. Those not clearly aligned were coded as “other.”

SILC‐MCHNs also used telephone calls and text messaging to contact and follow up women during the trial period. Telephone logs were kept separate from the home visit data collection forms; however, the logs were poorly completed and therefore considered unreliable. Analysis of these data was not undertaken.

Approval for the study was granted by the La Trobe University Human Ethics Committee (project number 11‐068) and the Department of Education and Early Childhood Development (project number 2011_001305). The methods used to conduct this study are described in detail in the trial protocol 13 and main outcome paper 25.

## Results

SILC‐MCHNs visited 1,043 women from September 2012 to March 2013. Most women (87%) receiving the home visit intervention were over 25 years of age, with the mean maternal age 30.5 years (SD 5.1) (Table 1). For 64 percent of women, this was their first baby. Overall, 67 percent of infants were seen within the first 14 days (average 10 days), with 18 percent being seen within the first week of life 14. Women visited were similar with regard to parity and maternal age across municipalities.

**Table 1:** Characteristics of Women Who Received a Home Visit for Breastfeeding Support, Victoria, Australia, 2012–2013

	Total *n* = 1,043	Mun 1 *n* = 164	Mun 2 *n* = 153	Mun 3 *n* = 53	Mun 4 *n* = 60	Mun 5 *n* = 251	Mun 6 *n* = 362
	*n* † (%)	*n* † (%)	*n* † (%)	*n* † (%)	*n* † (%)	*n* † (%)	*n* † (%)
Maternal age (years), mean ± SD	30.5 ± 5.1	29.7 ± 5.6	30.6 ± 5.7	30.0 ± 5.9	30.1 ± 5.2	30.7 ± 4.5	30.8 ± 4.7
Mothers aged < 25 years	128 (12.7)	33 (20.3)	30 (20.7)	10 (18.9)	7 (11.7)	22 (9.4)	26 (7.5)
Mothers aged 25–34 years	688 (68.5)	103 (63.2)	80 (55.2)	31 (58.5)	40 (66.7)	173 (73.6)	261 (74.8)
Mothers aged ≥ 35 years	189 (18.8)	27 (16.6)	35 (24.1)	12 (22.6)	13 (21.7)	40 (17.0)	62 (17.8)
Infant age (days), mean ± SD	16.4 **±** 18.0	14.9 **±** 24.0	18.7 **±** 17.0	23.1 **±** 17.6	9.1 **±** 3.4	11.8 **±** 8.6	19.4 **±** 20.3
Infants aged ≤ 7 days at 1st visit	190 (18.3)	43 (26.2)	14 (9.2)	5 (9.4)	22 (36.7)	64 (26.0)	42 (11.6)
Infants aged ≤ 14 days at 1st visit	690 (66.5)	126 (76.8)	90 (58.8)	17 (32.1)	53 (88.3)	201 (81.7)	203 (56.1)
Parity							
Primiparous	660 (64.1)	110 (67.9)	87 (57.2)	29 (54.7)	37 (61.7)	165 (67.6)	232 (64.8)
Multiparous	369 (35.9)	52 (32.1)	65 (42.8)	24 (45.3)	23 (38.3)	79 (32.4)	126 (35.2)
Feeding in the last 24 hours							
Fully breastfeeding	222 (21.5)	44 (27.0)	30 (19.6)	15 (28.3)	26 (44.1)	26 (10.6)	81 (22.6)
Breastfeeding and expressed breastmilk	200 (19.4)	50 (30.7)	39 (25.5)	9 (17.0)	13 (22.0)	29 (11.8)	60 (16.7)
Expressed breastmilk only	35 (3.4)	1 (0.6)	5 (3.3)	1 (1.9)	0 (0.0)	7 (2.9)	21 (5.9)
Breastfeeding and formula	167 (16.2)	7 (4.3)	15 (9.8)	9 (17.0)	2 (3.4)	72 (29.3)	62 (17.3)
Breastfeeding, expressed breastmilk, and formula	296 (28.7)	49 (30.1)	46 (30.1)	13 (24.5)	15 (25.4)	88 (35.8)	85 (23.7)
Expressed breastmilk and formula	93 (9.0)	9 (5.5)	16 (10.5)	3 (5.7)	2 (3.4)	20 (8.1)	43 (12.0)
Formula only*	20 (1.9)	3 (1.8)	2 (1.3)	3 (5.7)	1 (1.7)	4 (1.6)	7 (2.0)
Number of visits							
1 visit	762 (73.1)	108 (65.9)	99 (64.7)	34 (64.2)	48 (80.0)	209 (83.3)	264 (72.9)
2 visits	197 (18.9)	41 (25.0)	26 (17.0)	14 (26.4)	11 (18.3)	28 (11.2)	77 (21.3)
3 visits	60 (5.8)	12 (7.3)	18 (11.8)	5 (9.4)	1 (1.7)	5 (2.0)	19 (5.3)
4 or more visits	24 (2.3)	3 (1.8)	10 (6.5)	0 (0.0)	0 (0.0)	9 (3.6)	2 (0.6)
Visit duration (minutes), mean ±SD	66.8 ± 20.3	68.0 ± 18.4	71.9 ± 18.3	55.7 ± 23.1	67.7 ± 17.0	60.6 ± 17.0	69.5 ± 22.3

*Includes four women expressing and discarding breastmilk as a result of short‐term medication intake. ^†^Denotes full set of home visit logs received from each municipality. Some questions were incomplete but missing data were taken into account when calculating means and SDs. In total, there were a maximum total of 38 missing data points per question. One municipality was not missing any data. Of the others, missing data ranged from 1 (Municipality 4) to 20 (Municipality 5) data points per question. Mun = Municipality.

In all municipalities, SILC‐MCHNs typically spent 60–70 min with each woman during a home visit (Table 1). The number of visits each woman received from the SILC‐MCHN varied, with 73 percent receiving a single visit and 19 percent being seen twice. During the visits, SILC‐MCHNs responded to the individual circumstances of women, seeking to focus on what they identified as their most pressing breastfeeding issues. Many of these issues were addressed through reassurance (91%). This reassurance was often coupled with education about breastfeeding and discussions about expected newborn behaviors.

At the time of the home visit, 21.5 percent of infants were fully breastfeeding “in the past 24 hours”; this proportion ranged from 11 percent in Municipality 5 to 44 percent in Municipality 4 (Table 1). The majority of infants (61%) had received expressed breastmilk in the preceding 24 hours and 11% had received infant formula. There were a small number of women (*n* = 20) who had exclusively formula fed their babies in that time period. Four of these women indicated this was because they were taking medications and were expressing and discarding breastmilk to maintain supply, and planned to return to giving breastmilk to their infant once they were no longer taking the medication.

SILC‐MCHNs reported providing reassurance to 91 percent of women visited (Table 2). A high proportion were provided with information around the “mechanics” of breastfeeding, such as feeding frequency (78%), positioning and attachment (79%), and supply and demand (82%). In addition, “not enough milk” was further identified as the topic of discussion in 41 percent of visits. Normal infant behaviour was discussed with 57 percent of all women and 62 percent of first time mothers. Expressing breastmilk and nipple pain were also among the more common topics of discussion. More detail is provided in Table 2.

**Table 2:** Topics Discussed by SILC‐MCHN at First Home Visit, Victoria, Australia, 2012–2013

Topic discussed*	Total *n* = 1,043	First baby *n* = 660	Maternal age < 25 years *n* = 128	Infant age ≤ 7 days *n* = 190	Infant age ≤ 14 days *n* = 690
	*n* (%)	*n* (%)	*n* (%)	*n* (%)	*n* (%)
Reassurance	947 (90.8)	604 (91.5)	114 (89.1)	174 (91.6)	627 (90.9)
General breastfeeding information	863 (82.7)	572 (86.7)	110 (85.9)	160 (84.2)	581 (84.2)
Supply and demand	863 (82.7)	562 (85.2)	111 (86.7)	157 (82.6)	581 (84.2)
Positioning and attachment	821 (78.7)	537 (81.4)	94 (73.4)	158 (83.2)	560 (81.2)
Feed frequency	818 (78.4)	537 (81.4)	109 (85.2)	147 (77.4)	549 (79.6)
Normal infant behavior	591 (56.7)	408 (61.8)	72 (56.3)	90 (47.4)	372 (53.9)
Expressing	563 (54.0)	366 (55.5)	80 (62.5)	94 (49.5)	358 (51.9)
Nipple pain/damage	431 (41.3)	272 (41.2)	49 (38.3)	93 (49.0)	316 (45.8)
Not enough milk	428 (41.0)	286 (43.3)	51 (39.8)	67 (35.3)	272 (39.4)
Nipple shield	190 (18.2)	133 (20.2)	31 (24.2)	40 (21.1)	130 (18.8)
Mastitis	104 (10.0)	63 (9.6)	12 (9.4)	20 (10.5)	65 (9.4)
Maternal health problem	100 (9.6)	60 (9.1)	11 (8.6)	23 (12.1)	76 (11.0)
Oversupply	82 (7.9)	42 (6.4)	11 (8.6)	17 (9.0)	47 (6.8)
Tongue‐tie†	71 (6.8)	41 (6.2)	10 (7.8)	11 (5.8)	45 (6.5)
Maternal medication	65 (6.2)	44 (6.7)	8 (6.3)	7 (3.7)	45 (6.5)
Engorgement	53 (5.1)	34 (5.2)	5 (3.9)	16 (8.4)	37 (5.4)
Nipple/breast thrush	48 (4.6)	28 (4.2)	10 (7.8)	3 (1.6)	23 (3.3)
Effect of formula use on breastfeeding	31 (3.0)	19 (2.9)	10 (7.8)	3 (1.6)	10 (1.5)
Baby unwell	25 (2.4)	17 (2.6)	6 (4.7)	5 (2.6)	12 (1.7)
Jaundice	19 (1.8)	13 (2.0)	2 (1.6)	10 (5.3)	18 (2.6)
Reverse pressure softening	11 (1.1)	4 (0.6)	1 (0.8)	2 (1.1)	8 (1.2)
Breast refusal	10 (1.0)	9 (1.4)	1 (0.8)	0 (0.0)	4 (0.6)
Prematurity	9 (0.9)	6 (0.9)	1 (0.8)	2 (1.1)	6 (0.9)
Weaning and suppression	7 (0.7)	3 (0.5)	4 (3.1)	1 (0.5)	2 (0.3)
Baby's weight	6 (0.6)	3 (0.5)	0 (0.0)	0 (0.0)	0 (0.0)
Nipple vasospasm‡	5 (0.5)	2 (0.3)	1 (0.8)	1 (0.5)	3 (0.4)
Other(s)§	103 (9.9)	68 (10.3)	22 (17.2)	23 (12.1)	64 (9.3)

*Respondents could tick more than one option. ^†^Tongue‐tie (or ankyloglossia) is an abnormally short frenulum under the infant's tongue which restricts movement of the tongue and may lead to difficulty with attachment, nipple damage, and potentially a difficulty in establishing and maintaining milk supply. ^‡^Vasospasm is a spasm of the blood vessels of the nipple resulting in decreased blood flow to the nipple which may cause nipple and/or breast pain. ^§^Other topics included: Twins (*n* = 5); infant thrush (*n* = 4); returning to work (*n* = 4); alcohol and breastfeeding (*n* = 4); reflux (*n* = 4); blocked ducts (*n* = 4); postnatal depression and anxiety (*n* = 3); dummy/pacifier (*n* = 3); maternal diet (*n* = 3); social supports (*n* = 3); supply lines (*n* = 3). SILC‐MCHN = Supporting breastfeeding In Local Communities‐Maternal and Child Health Nurses.

Although the discussion topics recorded by the SILC‐MCHNs during the home visit were generally consistent across municipalities, one exception was the variation in how often nipple shields were discussed. While the topic of nipple shields was raised in 18 percent of visits, this ranged from a low of 3 percent (Municipality 5) to a high of 42 percent (Municipality 4) (data by municipality not shown).

Resources provided and/or recommended were similar across municipalities and included printed resources such as brochures and handouts, and information about websites (Tables 3 and 4). *Printed material* most commonly consisted of fact sheets from the Royal Women's Hospital, Melbourne on a range of issues, and from the Australian Breastfeeding Association (the main mother‐to‐mother support group in Australia) about expressing and low milk supply.

**Table 3:** Fact Sheets Provided by SILC‐MCHN During First Home Visit, Victoria, Australia, 2012–2013

Fact sheet provided*	Total *n* = 1,043
	*n* (%)
Expressing breastmilk†	276 (26.5)
Low milk supply†	200 (19.2)
Australian Breastfeeding Association brochures‡	154 (14.8)
Other RWH fact sheets†	
Breastfeeding: getting started	133 (12.8)
Domperidone for increasing breastmilk supply	119 (11.4)
Nipple shields	107 (10.3)
How to breastfeed	106 (10.2)
Using a breast pump	100 (9.6)
Australian Breastfeeding Association *Breastfeeding confidence* e‐book	88 (8.4)
Mastitis	59 (5.7)
Breastfeeding: supplementary feeds	43 (4.1)
Tongue‐tie	42 (4.0)
Breast and nipple thrush	40 (3.8)
Nipple vasospasm	20 (1.9)
Rebecca Glover *The Key to Successful Breastfeeding*	52 (5.0)
Preparing a bottle feed using baby milk powder (BFI‐UK)	12 (1.2)
Breast compression	5 (0.5)
Complementary and alternative medicines and breastfeeding	4 (0.4)
How common medications can affect your breastmilk	4 (0.4)
Sterilizing baby feeding equipment	4 (0.4)
Other(s)§	30 (2.9)

*Respondents could tick more than one option. ^†^Royal Women's Hospital fact sheets available from www.thewomens.org.au/atozfactsheets. ^‡^Covering a range of breastfeeding topics. ^§^Other fact sheets included: sleep and play for babies under 1 year (n = 4); and brochures for other services (*n* = 3). SILC‐MCHN = Supporting breastfeeding In Local Communities‐Maternal and Child Health Nurses; BFI‐UK = Baby Friendly Initiative−UK.

**Table 4:** Websites Recommended by SILC‐MCHN During the First Home Visit, Victoria, Australia, 2012–2013

Websites recommended*	Total *n* = 1,043	Mun 1 *n* = 164	Mun 2 *n* = 153	Mun 3 *n* = 53	Mun 4 *n* = 60	Mun 5 *n* = 251	Mun 6 *n* = 362
	*n* (%)	*n* (%)	*n* (%)	*n* (%)	*n* (%)	*n* (%)	*n* (%)
Australian Breastfeeding Association www.breastfeeding.asn.au	583 (55.9)	126 (76.8)	145 (94.8)	44 (83.0)	57 (95.0)	74 (29.5)	137 (37.9)
Royal Women's Hospital Fact sheets https://www.thewomens.org.au/health-information/breastfeeding/	475 (45.5)	128 (78.1)	145 (94.8)	44 (83.0)	57 (95.0)	79 (31.5)	22 (6.1)
Dr Jack Newman's website www.breastfeedinginc.ca	449 (43.1)	85 (51.8)	140 (91.5)	0 (0.0)	57 (95.0)	0 (0.0)	167 (46.1)
Raising Children Network http://raisingchildren.net.au	422 (40.5)	106 (64.6)	144 (94.1)	45 (84.9)	57 (95.0)	68 (27.1)	2 (0.6)
Caring for your baby at night (BFI‐UK) http://www.unicef.org.uk/BabyFriendly/Resources/Resources-for-parents/Caring-for-your-baby-at-night	249 (23.9)	0 (0.0)	141 (92.2)	0 (0.0)	56 (93.3)	0 (0.0)	52 (14.4)
Better Health Channel http://www.betterhealth.vic.gov.au	247 (23.7)	1 (0.6)	143 (93.5)	42 (79.3)	57 (95.0)	3 (1.2)	1 (0.3)
Breastfeeding information on Health Talk online www.healthtalk.org	246 (23.6)	46 (28.1)	142 (92.8)	0 (0.0)	57 (95.0)	0 (0.0)	1 (0.3)
Off to the best start (BFI‐UK) http://www.unicef.org.uk/BabyFriendly/Resources/Resources-for-parents/Off-to-the-best-start/	245 (23.5)	1 (0.6)	143 (93.5)	0 (0.0)	57 (95.0)	0 (0.0)	44 (12.2)
Alcohol and breastfeeding information (Australian Breastfeeding Association website) https://www.breastfeeding.asn.au/bf-info/safe-when-breastfeeding/alcohol-and-breastfeeding	213 (20.4)	2 (1.2)	142 (92.8)	2 (3.8)	57 (95.0)†	8 (3.2)	2 (0.6)
Other(s)‡	169 (16.2)	2 (1.2)	3 (2.0)	0 (0.0)	7 (11.7)	0 (0.0)	157 (43.4)

*Respondents could tick more than one option.^†^These SILC MCHNs used the same websites with almost all women. ^‡^Other websites included: YouTube (*n* = 36); Catherine Watson‐Genna's website (*n* = 29); Kellymom (*n* = 18); Rebecca Glover's website (*n* = 5); “What were we thinking?” (*n* = 1); Medela (*n* = 1).SILC‐MCHN = Supporting breastfeeding In Local Communities‐Maternal and Child Health Nurses; BFI‐UK = Baby Friendly Initiative‐UK.

*Websites* included peer‐reviewed and government‐supported sites such as the Australian Breastfeeding Association (56%), Raising Children Network (41%), and Better Health Channel (24%) (Table 4). Other frequently recommended sites included those that offer instructional videos or resources (e.g., Dr Jack Newman's website) and information websites such as The Baby Friendly Initiative‐UK. Experience‐sharing sites such as Health Talk Online (29%) were also recommended. It was evident that the frequency of recommending particular websites varied across municipalities, indicating that SILC‐MCHNs had preferences about the resources they used, and used those resources often (Table 4).

When necessary, SILC‐MCHNs referred women to other health or support services (Table 5). In total, 173 women (17%) were referred to one or more services. Referrals were most commonly made to breastfeeding support services (*n* = 89) and general medical practitioners (*n* = 59). The Australian Breastfeeding Association was also reported as a source of referral, although this may have been recommended as an agency which could be contacted for 24‐hour telephone support, rather than as a formal referral. Other referrals were made to medicine information services, private lactation consultants, and other health care providers. SILC‐MCHNs from three municipalities also noted using interpreters and translated information for some families.

**Table 5:** Referrals Made by SILC‐MCHN During the First Home Visit, Victoria, Australia, 2012–2013

Referrals made*	Total *n* = 1,043
	*n* (%)
Breastfeeding service	89 (8.5)
General practitioner	59 (5.7)
Australian Breastfeeding Association	44 (4.2)
Service for information on medications (e.g. pharmacists, drug information services)	10 (1.0)
Enhanced MCH services	4 (0.4)
SILC drop‐in centers	3 (0.3)
Private lactation consultant	2 (0.2)
Emergency department	2 (0.2)
Perinatal emotional care services	2 (0.2)
Other(s)†	17 (1.6)

*Respondents could tick more than one option. ^†^Other referrals included: Physiotherapist (*n* = 1); chiropractor (*n* = 1); pediatrician (*n* = 1). SILC‐MCHN = Supporting breastfeeding In Local Communities‐Maternal and Child Health Nurses.

## Discussion

Overall, the SILC home visit data indicate that the SILC‐MCHNs provided breastfeeding support and reassurance in the early postpartum period to the majority of women they visited, irrespective of whether this was their first or subsequent child. This general reassurance was in addition to information on breastfeeding and a range of specific breastfeeding issues. Although reassurance is frequently mentioned in reports of other breastfeeding interventions 16, 23, 26, these papers are usually reporting on women who have sought breastfeeding support and are not inclusive of the breastfeeding population as a whole. The SILC home visits captured a wider population of breastfeeding mothers by providing proactive and preventative support for women at risk of ceasing breastfeeding, instead of targeting only women who asked for help with breastfeeding, which will make the findings applicable to a broader population.

The SILC‐MCHNs also frequently provided information relating to the “mechanics of breastfeeding” such as supply and demand, positioning, and feeding frequency, as well as normal newborn behavior. There is an increasing recognition of the need for women to understand typical newborn behaviors, especially to protect and support the breastfeeding relationship 5, 27 as these are commonly linked. A British study found a high level of misunderstanding of typical infant behavior and women frequently blamed the method of feeding for behaviors such as crying, wakefulness, and cluster feeding 28. Education in the early postpartum period has been shown to contribute to increased breastfeeding rates and duration, particularly if focussed on individual maternal needs 5, 29. The need for greater understanding by women of what to expect while breastfeeding was indicated by the provision of information about typical infant behaviors to the majority of women, whether or not this was their first baby.

Although education is an important element to encourage continued breastfeeding, it should be realistic, and an overly rigid educational approach may be detrimental to its success 23, 30. Studies exploring maternal perceptions of breastfeeding support have found that while support for ongoing breastfeeding was valued, it was important to recognize the competing priorities women face which influence their ability or desire to exclusively breastfeed their infants 23, 31. Early proactive care, targeting women at risk of stopping breastfeeding or introducing formula or solids, is thought to make a difference to breastfeeding duration and rates 23. A family‐centered approach which allowed for the development of individualized care plans was recommended in the literature to have the most beneficial influence on breastfeeding rates by building confidence and self‐efficacy through reassurance and guidance and allowing for a variety of breastfeeding experiences 23, 30, 31. The SILC intervention aimed to adopt this approach and the SILC‐MCHNs provided guidance through family‐centered, proactive care to women most likely to cease breastfeeding.

Women face many issues that affect their capacity to initiate and maintain the breastfeeding relationship. Nipple pain, perceived low supply, and expressing (pumping) of breastmilk are known to negatively affect breastfeeding duration 32, 33, 34. Pain and low supply have also been linked with an increased likelihood of women experiencing symptoms of anxiety and depression 8. This study reinforced that these were important issues experienced by a significant number of women in the community, not only those seeking breastfeeding support.

Our data indicated that concern about milk supply was common and that supply was discussed with almost half the women visited. In one study of 1,000 women from the United States, 60 percent of women indicated they ceased breastfeeding earlier than planned, predominantly as a result of concerns relating to difficulties with breastfeeding or expressing milk for their infant, and infant growth 35. A number of other researchers have also found that perceived low supply is the main reason for women reducing or ceasing breastfeeds 33, 36, as well as for expressing (pumping) breastmilk 18, 33, 36 and bottle feeding 37. Clinicians should be aware of the common misconceptions about adequacy of milk supply and actively dispel these when appropriate because breastfeeding problems have been linked to an increased risk of postnatal depression 8.

It is worth also noting that one of the breastfeeding topics discussed at almost one in five home visits was the use of nipple shields. Nipple shields are designed for use by women with inverted nipples, premature infants, or when other physical factors make attachment at the breast challenging 38. They are recommended as a short‐term strategy to maintain the breastfeeding relationship until the infant can attach directly to the breast, and are best managed with input from experienced, professional breastfeeding support. If nipple shields are used by women as a treatment for nipple pain without ongoing breastfeeding guidance, there is increased likelihood that nipple shield use may affect infant growth and reduce the duration of breastfeeding 32, likely as a result of reduced milk transfer compared with direct breastfeeding 39.

This documentation of breastfeeding support in the community confirms that experienced health professionals can provide important home‐based education and support to women in the early weeks after discharge from the maternity services. The strength of this paper is a broader perspective of these issues in comparison to studies of women seeking help with breastfeeding. The activities carried out by the SILC‐MCHNs were consistent across municipalities and were delivered in accordance with the expectations of the study design 13. Over 1,000 women were visited at home and data about the content of the visit were systematically recorded. A limitation of the study is that the data were recorded by the SILC‐MCHNs on precoded data sheets which simplified data collection, but did not provide us with a deep understanding of the reasons the nurses selected particular data items.

### Conclusion and Recommendations

This study explored the concerns and care of women at risk of breastfeeding cessation in entire communities in Victoria, Australia. It also explored the content of community home‐based support in the early postpartum period. Given this study was representative of the breastfeeding population as a whole, these findings are likely to be generalizable, at least to municipalities with similar characteristics, and probably beyond.

Key aspects of this support were reassurance and normalization of infant behaviors through a family‐centered approach. As expected, the SILC‐MCHNs provided information to most women about the basic mechanics of breastfeeding (positioning and attachment to the breast, supply and demand) as this information is essential for breastfeeding success. We also found that individual clinicians have personal and location‐based preferences for the resources they use and tend to use these with most families. This highlights a need for these resources to be of a high standard, up to date, and appropriately applied in each community.

## Funding

Department of Education and Early Childhood Development (now Department of Education and Training), Victoria, Australia

## Trial registration

Australian New Zealand Clinical Trials Registry ACTRN12611000898954

## Supporting information


**Appendix 1.** Documentation of Home Visit for SILC Trial, Victoria, Australia, 2012–2013.

## References

[1] Amir LH , Donath SM . Socioeconomic status and rates of breastfeeding in Australia: Evidence from three recent national health surveys. Med J Aust 2008;189(5):254–256. 18759719 10.5694/j.1326-5377.2008.tb02016.x

[2] Australian Institute of Health and Welfare (AIHW) . 2010 Australian national infant feeding survey: Indicator results. Cat. no. PHE 156. Canberra: AIHW; 2011.

[3] Victorian Government Department of Education and Training (DET) . Maternal and child health services annual report (Statewide) 2014–2015. 2015.

[4] Kronborg H , Harder I , Hall EOC . First time mothers' experiences of breastfeeding their newborn. Sex Reprod Healthc 2015;6(2):82–87. 25998875 10.1016/j.srhc.2014.08.004

[5] Renfrew MJ , McCormick FM , Wade A , et al. Support for healthy breastfeeding mothers with healthy term babies. Cochrane Database Syst Rev 2012;5:CD001141. 10.1002/14651858.CD001141.pub4 PMC3966266 22592675

[6] State Government of Victoria . Victorian breastfeeding plan: Supporting, promoting and protecting breastfeeding. 2014.

[7] Baxter J , Cooklin AR , Smith J . Which mothers wean their babies prematurely from full breastfeeding? An Australian cohort study Acta Paediatr 2009;98(8):1274–1277. 19469772 10.1111/j.1651-2227.2009.01335.x

[8] Brown A , Rance J , Bennett P . Understanding the relationship between breastfeeding and postnatal depression: The role of pain and physical difficulties. J Adv Nurs 2016;72(2):273–282. 26494433 10.1111/jan.12832 PMC4738467

[9] United Nations Childrens Fund (UNICEF) . Breastfeeding on the worldwide agenda: Findings from a landscape analysis on political commitment for programmes to protect, promote and support breastfeeding. UNICEF, 2013.

[10] Victorian Government Department of Education and Early Childhood Development (DEECD) and Municipal Association Victoria . Maternal and child health service program standards. Melbourne: Office for Children; 2009.

[11] National Health and Medical Research Council (NHMRC) . Infant feeding guidelines: Information for health workers (2012). Canberra Department of Health and Ageing; 2012.

[12] Department of Education and Early Childhood Development (DEECD) . Promoting breastfeeding: Victorian breastfeeding guidelines. Melbourne: DEECD; 2014.

[13] McLachlan HL , Forster DA , Amir LH , et al. Supporting breastfeeding in local communities (SILC): Protocol for a cluster randomised controlled trial. BMC Pregnancy Childbirth 2014;14:346. 25281300 10.1186/1471-2393-14-346 PMC4287548

[14] Chin LY , Amir LH . Survey of patient satisfaction with the Breastfeeding Education and Support Services of The Royal Women's Hospital, Melbourne. BMC Health Serv Res 2008;8:83. 18405394 10.1186/1472-6963-8-83 PMC2335099

[15] Coffield K . The benefits of phone support and home visits: An evaluation of the City of Kingston's breastfeeding support service. Breastfeed Rev 2008;16(3):17–21. 19133399

[16] Cowie GA , Hill S , Robinson P . Using an online service for breastfeeding support: What mothers want to discuss. Health Promot J Austr 2011;22(2):113–118. 21819353 10.1071/he11113

[17] Avery A , Zimmermann K , Underwood PW , Magnus JH . Confident commitment is a key factor for sustained breastfeeding. Birth 2009;36(2):141–148. 19489808 10.1111/j.1523-536X.2009.00312.x

[18] Forster DA , Johns HM , McLachlan HL , et al. Feeding infants directly at the breast during the postpartum hospital stay is associated with increased breastfeeding at 6 months postpartum: A prospective cohort study. BMJ Open 2015;5(5:e006571). 10.1136/bmjopen-2014-007512 PMC4431142 25953728

[19] Victorian Government Department of Education and Early Childhood Development (DEECD) . Maternal and child health services annual report (South‐Western Victoria Region) 2012‐2013. 2013.

[20] Victorian Government Department of Education and Early Childhood Development (DEECD) . Maternal and child health services annual report (North‐Western Victoria Region) 2012‐2013. 2013.

[21] Victorian Government Department of Education and Early Childhood Development (DEECD) . Maternal and child health services annual report (South‐Eastern Victoria Region) 2012‐2013. 2013.

[22] Victorian Government Department of Education and Early Childhood Development (DEECD) . Maternal and child health services annual report (North‐Eastern Victoria Region) 2012‐2013. 2013.

[23] Hoddinott P , Craig LCA , Britten J , McInnes RM . A serial qualitative interview study of infant feeding experiences: Idealism meets realism. BMJ Open 2012;2(2):e000504. 10.1136/bmjopen-2011-000504 PMC3307036 22422915

[24] StataCorp . Stata Statistical Software. Release 12. College Station, TX: StataCorp LP; 2011.

[25] McLachlan HL , Forster DA , Amir LH , et al. Supporting breastfeeding in local communities (SILC) in Victoria, Australia: A cluster randomised controlled trial. BMJ Open 2015;6(2):e008292. 10.1136/bmjopen-2015-008292 PMC4746449 26832427

[26] Gill SL , Reifsnider E , Lucke JF . Effects of support on the initiation and duration of breastfeeding. West J Nurs Res 2007;29(6):708–723. 17557933 10.1177/0193945906297376

[27] Brown A , Harries V . Infant sleep and night feeding patterns during later infancy: Association with breastfeeding frequency, daytime complementary food intake, and infant weight. Breastfeed Med 2015;10(5):246–252. 25973527 10.1089/bfm.2014.0153

[28] Spencer R , Greatrex‐White S , Fraser DM . ‘I was meant to be able to do this’: A phenomenological study of women's experiences of breastfeeding. Evidence Based Midwifery 2014;12(3):83–88.

[29] Brenner MG , Buescher ES . Breastfeeding: A clinical imperative. J Women's Health (15409996) 2011;20(12):1767–1773. 10.1089/jwh.2010.2616 21967047

[30] Schmied V , Beake S , Sheehan A , et al. Women's perceptions and experiences of breastfeeding support: A metasynthesis. Birth 2011;38(1):49–60. 21332775 10.1111/j.1523-536X.2010.00446.x

[31] MacVicar S , Kirkpatrick P , Humphrey T , Forbes‐McKay KE . Supporting breastfeeding establishment among socially disadvantaged women: A meta‐synthesis. Birth 2015;42(4):290–298. 26255973 10.1111/birt.12180

[32] Ekstrom A , Abrahamsson H , Eriksson RM , Martensson BL . Women's use of nipple shields‐Their influence on breastfeeding duration after a process‐oriented education for health professionals. Breastfeed Med 2014;9(9):458–466. 25188544 10.1089/bfm.2014.0026

[33] Li R , Fein SB , Chen J , Grummer‐Strawn LM . Why mothers stop breastfeeding: Mothers' self‐reported reasons for stopping during the first year. Pediatrics 2008;122(Supplement 2):S69–S76. 18829834 10.1542/peds.2008-1315i

[34] Buck ML , Amir LH , Cullinane M , Donath SM . CASTLE Study Team. Nipple pain, damage, and vasospasm in the first 8 weeks postpartum. Breastfeed Med 2014;9(2):56–62. 24380583 10.1089/bfm.2013.0106 PMC3934541

[35] Odom EC , Li R , Scanlon KS , et al. Reasons for earlier than desired cessation of breastfeeding. Pediatrics 2013;131(3):e726–e732. 23420922 10.1542/peds.2012-1295 PMC4861949

[36] Amir LH , Cwikel J . Why do women stop breastfeeding? A closer look at ‘not enough milk’ among Israeli women in the Negev region. Breastfeed Rev 2005;13(3):7–13. 16342407

[37] Rigotti RR , de Oliveira MIC , Boccolini CS . Association between the use of a baby's bottle and pacifier and the absence of breastfeeding in the second six months of life. Ciência & Saúde Coletiva 2015;20:1235–1244. 25923634 10.1590/1413-81232015204.00782014

[38] Watson Genna C . Nipple Shields. Selecting and Using Breastfeeding Tools: Improving Care and Outcomes. Amarillo, TX: Hale Publishing; 2009 p. 49–64.

[39] McKechnie AC , Eglash A . Nipple shields: A review of the literature. Breastfeeding Med. 2010;5(6):309–314. 10.1089/bfm.2010.0003 PMC3014757 20807104

