# Supplementary material for: Breastfeeding Support in the Early Postpartum: Content of Home Visits in the SILC Trial
Source: Birth. 2016 Jul 15;43(4):303–12. doi: 10.1111/birt.12241 (PMC5248617; doi:10.1111/birt.12241)
Supplement: Supplementary file 1 — Appendix 1. Documentation of Home Visit for SILC Trial, Victoria, Australia, 2012–2013. [file BIRT-43-303-s001.docx]

| **Appendix 1: Documentation of home visit for SILC trial, Victoria, Australia, 2012-2013** | | | | | | | | |
| --- | --- | --- | --- | --- | --- | --- | --- | --- |
| 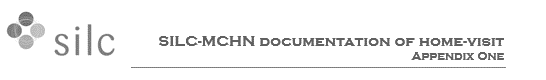 Name of Local Government Area: | | | | | | | | |
| **First two letters of mother’s first name** | | |  | **Referral pathway** | Initial SILC identification phone call  Follow-up (i.e. subsequent home visit)  Other referral (e.g. MCHN, drop-in centre) | | | |
| **First two letters of mother’s surname** | | |  |  |  |  |  |  |
| **Mother’s DOB** | | | ___/____/______ | **Date** | ___/____/______ | | | |
| **Baby’s DOB** | | | ___/____/______ | **Duration of visit (mins)** | ____________ | | | |
| **First live baby** | | | Yes  No |  |  |  |  |  |
|  |  | | | | | | |  |
| **1** | **In the last 24 hours, what feeds has your baby had? (tick only one)** | | | | | | |  |
|  | _1_ | Fully breastfeeding at the breast (i.e. no EBM, no formula) | | | | | |  |
|  | _2_ | Breastfeeding + EBM | | | | | |  |
|  | _3_ | EBM only | | | | | |  |
|  | _4_ | Breastfeeding + formula | | | | | |  |
|  | _5_ | Breastfeeding + EBM + formula | | | | | |  |
|  | _6_ | EBM + formula | | | | | |  |
|  | _7_ | Formula only | | | | | |  |
|  | _8_ | Other _______________________________________________ | | | | | |  |
| **2** | | **Topics discussed (tick all that apply)** | | | | |  |  |
|  | | _1_ | General breastfeeding information | | | |  |  |
|  | | _2_ | Reassurance | | | |  |  |
|  | | _3_ | Supply and demand | | | |  |  |
|  | | _4_ | Feed frequency | | | |  |  |
|  | | _5_ | Positioning and attachment | | | |  |  |
|  | | _6_ | Normal infant behaviour | | | |  |  |
|  | | _7_ | Nipple pain / damage | | | |  |  |
|  | | _8_ | Not enough milk | | | |  |  |
|  | | _9_ | Oversupply | | | |  |  |
|  | | _10_ | Engorgement | | | |  |  |
|  | | _11_ | Mastitis | | | |  |  |
|  | | _12_ | Nipple / breast thrush | | | |  |  |
|  | | _13_ | Expressing | | | |  |  |
|  | | _14_ | Nipple shield | | | |  |  |
|  | | _15_ | Tongue-tie | | | |  |  |
|  | | _16_ | Baby unwell | | | |  |  |
|  | | _17_ | Maternal health problem | | | |  |  |
|  | | _18_ | Maternal medication issue | | | |  |  |
|  | | _19_ | Other(s)_________________________________________________________________ | | | |  |  |
| **3** | | **Factsheet(s) provided from** [www.thewomens.org.au/atozfactsheets](http://www.thewomens.org.au/atozfactsheets) **(tick all that apply)** | | | | |  |  |
|  | | _1_ | Breastfeeding: getting started | | | |  |  |
|  | | _2_ | How to breastfeed | | | |  |  |
|  | | _3_ | Low milk supply | | | |  |  |
|  | | 4 | Domperidone for increasing breastmilk supply | | | |  |  |
|  | | 5 | Expressing breastmilk | | | |  |  |
|  | | 6 | Using a breast pump | | | |  |  |
|  | | 7 | Nipple vasospasm | | | |  |  |
|  | | 8 | Breast and nipple thrush | | | |  |  |
|  | | 9 | Nipple shields | | | |  |  |
|  | | 10 | Mastitis | | | |  |  |
|  | | 11 | Tongue-tie | | | |  |  |
|  | | 12 | Complementary and alternative medicines and breastfeeding | | | |  |  |
|  | | 13 | How common medications can affect your breastmilk | | | |  |  |
|  | | 14 | Breastfeeding: supplementary feeds | | | |  |  |
|  | | 15 | Preparing a bottle feed using baby milk powder (BFI-UK) | | | |  |  |
|  | | 16 | Sterilising baby feeding equipment | | | |  |  |
|  | | 17 | ABA Breastfeeding Confidence e-book | | | |  |  |
|  | | 18 | Other(s)_________________________________________________________________ | | | |  |  |
| **4** | | **Website information provided (tick all that apply)** | | | | |  |  |
|  | | _18_ | Raising Children Network  <http://raisingchildren.net.au/> | | | |  |  |
|  | | _19_ | Royal Women’s Hospital Factsheets  [www.thewomens.org.au/atozfactsheets](http://www.thewomens.org.au/atozfactsheets) | | | |  |  |
|  | | _20_ | Australian Breastfeeding Association website  <https://www.breastfeeding.asn.au/> | | | |  |  |
|  | | _21_ | Alcohol and breastfeeding information from the ABA website  <https://www.breastfeeding.asn.au/bf-info/safe-when-breastfeeding/alcohol-and-breastfeeding> | | | |  |  |
|  | | _22_ | Better Health Channel  <http://www.betterhealth.vic.gov.au/> | | | |  |  |
|  | | _23_ | Breastfeeding Information on Health Talk online  <http://www.healthtalkonline.org/Pregnancy_children/Breastfeeding> | | | |  |  |
|  | | _24_ | Off to the best start (BFI-UK) <http://www.unicef.org.uk/Documents/Baby_Friendly/Leaflets/4/otbs_leaflet.pdf> | | | |  |  |
|  | | _25_ | Caring for your baby at night (BFI-UK)  <http://www.unicef.org.uk/Documents/Baby_Friendly/Leaflets/caringatnight_web.pdf> | | | |  |  |
|  | | _26_ | Jack Newman's website  <http://www.breastfeedinginc.ca/index.php> | | | |  |  |
|  | | _27_ | Other(s) _______________________________________________________________ | | | |  |  |
| **5** | | **Referral to** | | | | | |  |
|  | | _1_ | GP | | | | |  |
|  | | _2_ | Private LC | | | | |  |
|  | | _3_ | ABA | | | | |  |
|  | | _4_ | Emergency department | | | | |  |
|  | | _5_ | Breastfeeding services ______________________________________________________ | | | | |  |
|  | | _6_ | Other(s)__________________________________________________________________ | | | | |  |
| **6** | | **Other (notes)** | | | | |  |  |
